# Comprehensive assessment of myocardial remodeling in ischemic heart disease by synchrotron propagation based X-ray phase contrast imaging

**DOI:** 10.1038/s41598-021-93054-6

**Published:** 2021-07-07

**Authors:** Ivo Planinc, Patricia Garcia-Canadilla, Hector Dejea, Ivana Ilic, Eduard Guasch, Monica Zamora, Fàtima Crispi, Marco Stampanoni, Davor Milicic, Bart Bijnens, Anne Bonnin, Maja Cikes

**Affiliations:** 1 University of Zagreb School of Medicine, Department of Cardiovascular Diseases, University Hospital Centre Zagreb, Kispaticeva 12, 10000 Zagreb, Croatia; 2 Institut d’Investigacions Biomèdiques August Pi i Sunyer (IDIBAPS), Barcelona, Spain; 3 BCNatal, Hospital Clínic and Hospital Sant Joan de Déu, Barcelona, Spain; 4 Paul Scherrer Institut, Villigen, Switzerland; 5 Institute for Biomedical Engineering, ETH Zürich, Zürich, Switzerland; 6 University of Zagreb School of Medicine, Department of Pathology, University Hospital Centre Zagreb, Zagreb, Croatia; 7 Arrhythmia Unit, Department of Cardiology, Hospital Clínic de Barcelona, Barcelona, Spain; 8 Centre for Biomedical Research on Cardiovascular Diseases (CIBER-CV), Madrid, Spain; 9 Hospital Clínic, Centre for Biomedical Research on Rare Diseases (CIBER-ER), Barcelona, Spain; 10 ICREA, Barcelona, Spain

**Keywords:** Myocardial infarction, Imaging techniques, Experimental models of disease, Biomedical engineering

## Abstract

Cardiovascular research is in an ongoing quest for a superior imaging method to integrate gross-anatomical information with microanatomy, combined with quantifiable parameters of cardiac structure. In recent years, synchrotron radiation-based X-ray Phase Contrast Imaging (X-PCI) has been extensively used to characterize soft tissue in detail. The objective was to use X-PCI to comprehensively quantify ischemic remodeling of different myocardial structures, from cell to organ level, in a rat model of myocardial infarction. Myocardial infarction-induced remodeling was recreated in a well-established rodent model. Ex vivo rodent hearts were imaged by propagation based X-PCI using two configurations resulting in 5.8 µm and 0.65 µm effective pixel size images. The acquired datasets were used for a comprehensive assessment of macrostructural changes including the whole heart and vascular tree morphology, and quantification of left ventricular myocardial thickness, mass, volume, and organization. On the meso-scale, tissue characteristics were explored and compared with histopathological methods, while microstructural changes were quantified by segmentation of cardiomyocytes and calculation of cross-sectional areas. Propagation based X-PCI provides detailed visualization and quantification of morphological changes on whole organ, tissue, vascular as well as individual cellular level of the ex vivo heart, with a single, non-destructive 3D imaging modality.

## Introduction

While ischemic heart disease is one of the most studied cardiac pathologies, many questions remain regarding the understanding of myocardial remodeling with improved treatment and more tissue salvage as the ultimate goal. To develop protective or tissue repair strategies, the in-depth understanding of the underlying pathophysiological processes and the involved structural changes of the heart are of paramount importance. Ideally this is amalgamated in an imaging modality enabling the integration of structural information from cell to organ level.

Cardiac remodeling is a combined response of cardiomyocytes and other cells, myocardial tissue, and the heart as an organ, to different adverse pathophysiological processes (such as loss of contractile force by necrosis or apoptosis of cardiomyocytes, cellular hypertrophy, intercellular matrix changes like fibrosis, or deposition of different metabolic end-products)^1^. These processes may result in changes in cardiac size, myocardial mass, (segmental) geometry, cardiomyocytes appearance and orientation, and ultimately function of the whole organ, thus having a profound impact on patient outcome. One of the typical examples of negative cardiac remodeling is induced by ischemic heart disease, with a myocardial infarction (MI) being the trigger of an adverse cascade. Major insights in the remodeling processes have been gained from animal models of cardiovascular disease (either rodents, or large animals such as sheep and swine) where using ex vivo gross anatomical morphology or histology have improved our knowledge of the induced changes^2–5^.

In clinical practice, ischemic heart disease is assessed by means of diagnostic imaging modalities, encompassing those that visualize the coronary arteries, but also that quantify the resulting adverse remodeling: coronary angiography, echocardiography, magnetic resonance imaging, computed tomography, and nuclear cardiology imaging are often combined as complementary techniques. Histopathological analysis is seldom utilized in routine clinical assessment of myocardial remodeling, but remains a standard research tool for detailing tissue and cell structure, while additional methods of histochemical staining help differentiate the expression of specific cellular or extracellular signals in tissues. Cardiovascular research, as well as clinical cardiology, are in an ongoing quest for a superior (imaging) method that could integrate gross-anatomical information with microanatomy, combined with quantifiable parameters of cardiac structure.

In recent years, synchrotron radiation-based X-ray Phase Contrast Imaging (X-PCI) has been extensively used to characterize soft tissue in detail. Synchrotrons are large scale facilities that generate highly collimated and brilliant X-ray beams. By exploiting the refractive properties of these beams in dedicated setups, X-PCI is able to image soft tissues or organs non-destructively in 3D and at high-resolution (down to < 1 µm), with increased contrast in comparison to conventional absorption imaging. Particularly in the cardiovascular field, X-PCI has so far been utilized for ex vivo imaging of animal models, human fetus hearts and transmural samples of adult human hearts, both normal and diseased^6–16^.

The aim of our study was to use X-PCI to comprehensively quantify remodeling of different myocardial structures, from cell to organ level, as a consequence of MI in a rat model.

## Results

### Macroscale

#### Whole heart morphology—quantification of LV myocardial thickness, mass and volume

**Figure 1:**
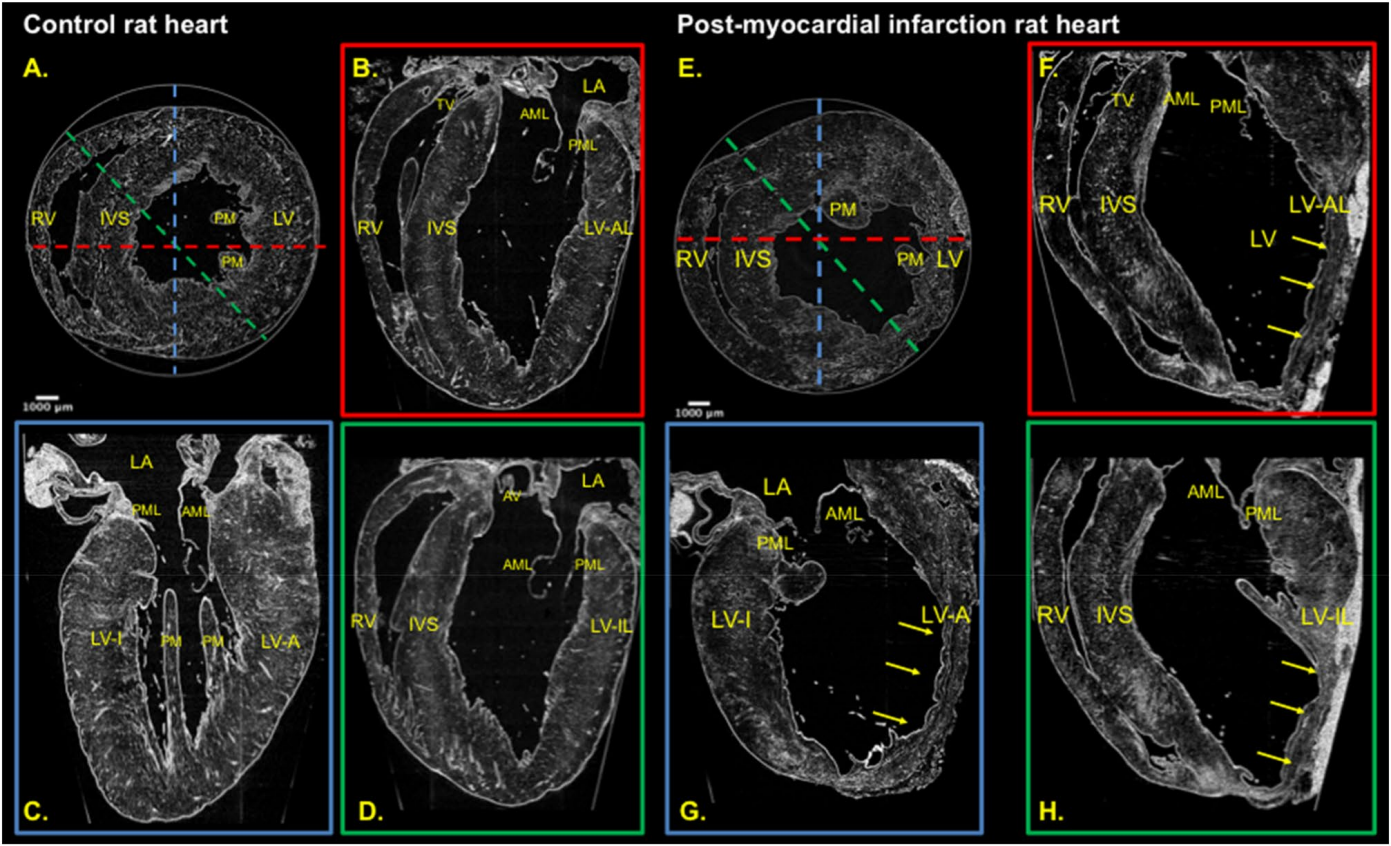
PB X-PCI images reconstructed as conventional imaging views. Control heart from one healthy rat was used for left panel images, and infarcted heart from rat with induced myocardial infarction was used for the right panel. Left panel—control; right panel—infarct. (**A**) Short axis view, (**B**) Apical 4 chamber view, (**C**) Apical 2 chamber view, (**D**) Apical 5 chamber view; (**E**) Short axis view, (**F**) Apical 4 chamber view, (**G**) Apical 2 chamber view, (**H**) Apical 5 chamber view. The dashed lines indicate the position of the slice perpendicular to the short axis, producing a specific longitudinal view. Short yellow arrows indicate the infarct areas with clear aneurysm formation. LV: left ventricle, RV: right ventricle, PM: papillary muscle, IVS: interventricular septum, PML: posterior leaflet of mitral valve, AML: anterior leaflet of mitral valve, TV: tricuspid valve, LA: left atrium, AV: aortic valve, LV-AL: anterolateral wall of the left ventricle, LV-A: anterior wall of the left ventricle, LV-I: inferior wall of the left ventricle, LV-IL: inferolateral wall of the left ventricle.

The obtained 3D datasets can be easily used to assess the gross anatomical features of imaged hearts. In Fig. 1, the low resolution (LR) propagation based (PB) X-PCI datasets were re-sliced to obtain the conventional imaging views, allowing clear visualization of the cardiac chambers, identification of the segments of the left ventricle (LV) and right ventricle (RV), as well as the different valve leaflets and details of the trabeculations. The whole heart acquisitions allow to clearly appreciate the exact extension of myocardial scar: in the right panel of Fig. 1, the infarct extends through most of the anterior, anterolateral and inferolateral LV walls, indicating a very large perfusion area of the ligated left coronary artery.

Supplementary Figure S1 shows an example of the 45-segment model of one of the infarcted hearts indicating the normal (contralateral), peri-, and MI area.

When quantifying LV cavity volumes, myocardial mass and wall thickness; thinner walls, larger cavity volumes and increased myocardial mass were observed in the infarcted hearts as compared to the control rat heart (Table 1 and Supplementary Figure S2).

**Table 1:** Parameters and quantification of global and cellular LV remodeling obtained by PB X-PCI imaging.

Myocardial (global) remodeling						
	Control rat heart	Post MI average	MI Heart 1	MI Heart 2	MI Heart 3	MI Heart 4
Wall thickness (mm)	**2.2 ± 0.4**	1.8 ± 0.8 (p = 0.028)*	1.9 ± 0.9	2.1 ± 0.9	1.8 ± 0.7	1.5 ± 0.7
LV cavity volume (ml)	**0.11**	0.26 ± 0.09 (p = 0.03)*	0.20	0.20	0.23	0.39
LV myocardial volume (ml)	**0.58**	0.62 ± 0.02 (p = 0.01)*	0.62	0.63	0.60	0.63
LV myocardial mass (g)	**0.61**	0.67 ± 0.03 (p = 0.02)*	0.71	0.67	0.63	0.66

Cellular remodeling						
Cardiomyocyte cross sectional area (CSA) (μm^2^)	Control rat heart	Post MI average	MI heart 1	MI heart 2		
Septum	**311** **(275–318)**	**459 (410–530)** **p = 0.0091**†	463 (413–479)	454 (416–550)		
Anterior wall	**296** **(285–322)**	**764 (640–810)** **p = 0.0021**†	778 (705–842)	729 (624–790)		
Overall	**304** **(278–321)**	**598 (461–776)** **p = 0.0001**†	609 (464–775)	592 (455–776)		

Normally distributed continuous variables are presented as means with standard deviations, and non-normally distributed as medians with interquartile range. Upper panel: LV wall thickness is averaged over 45 segments for each animal. Lower panel: CSA is averaged from 10 cardiomyocytes per region.

*LV* left ventricle, *MI* myocardial infarction.

*Comparisons of means between post-MI hearts (N = 4) and the healthy heart (N = 1) with a two-tailed Student's T test.

^†^Significant difference in CSA overall and between similar myocardial areas of affected and non-affected hearts (post-MI hearts N = 2, healthy heart N = 1), compared by a Kruskal Wallis non-parametric test.

#### Quantification of myocardial organization

**Figure 2:**
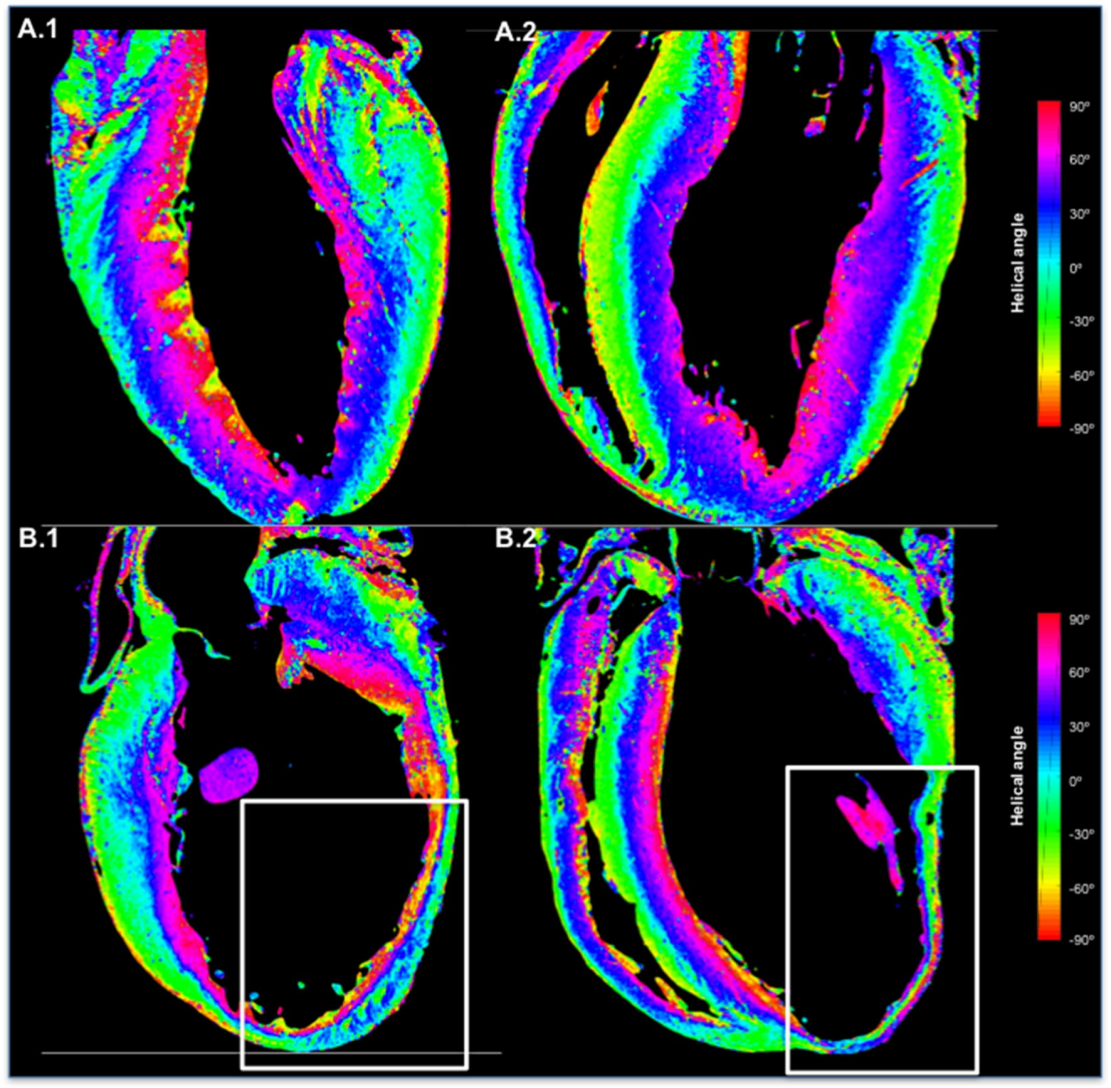
Longitudinal visualization of the local helical angles (HA) of myocyte aggregates. One control heart from a healthy rat, and one infarcted heart from a rat with induced myocardial infarction was used. Different colors represent different HAs. (**A.1**) Apical 2 chamber view, and (**A.2**) Apical 4 chamber view of the control heart. (**B.1**) Apical 2 chamber view of the infarcted heart showing a large aneurysm of the anterior wall (white rectangle). (**B.2**) Apical 4 chamber view of the same infarcted heart showing the apical extend of the aneurism (white rectangle).

For further insight into the structural changes occurring as a consequence of MI, we have quantified local cardiomyocyte aggregates orientation. A longitudinal view of the local helical angles (HA) values of an infarcted and control rat heart is shown in Fig. 2.

As expected, the HAs in control heart show predominance of positive angles in the endocardium with a smooth progression to negative angles when approaching the epicardium. A similar pattern of cardiomyocyte organization can be seen in the non-affected segments of the post-MI heart. In the infarct zone, the HA still show the ‘normal’ positive values at the endocardium (purple/dark blue color in Fig. 2), and negative values (green/yellow) at the epicardium, even in the segments of clear transmural scar. Preservation of the general cardiomyocyte orientation indicates certain amount of preserved cardiomyocyte aggregates in the endocardial and epicardial areas of the infarct zone. The results of linear fitting of the transmural HA for LV segments are shown in Supplementary Table S1. Also, the local HAs corresponding to the overall cardiomyocyte aggregate orientation are shown in Supplementary Figure S3.

Furthermore, the analysis of myocardial disarray index (MDI) shows clear differences in the local cardiomyocytes organization between different regions of the post-MI heart, as well as between the control rat heart and post-MI hearts in general (Table 2). MI segments have the lowest values of MDI indicating high local myocardial disarray. Also, all segments of the post-MI rat hearts have lower MDI values (i.e. more disarray) in comparison to the control rat heart (precisely, significantly lower for MI, peri-MI, and remaining segments, and borderline significant for contralateral segments).

**Table 2:** Myocardial disarray index (MDI) in different regions of all hearts. MDI is calculated for 45 segments in each heart. Significant differences are observed in MDI between post-MI hearts (N = 4) and control heart (N = 1), as well as between different regions of post-MI hearts.

Myocardial disarray index (MDI)						
	Control	Post-MI	MI heart 1	MI heart 2	MI heart 3	MI heart 4
MI segments	**–**	**0.77** **(0.72–0.82)**	0.73 (0.70–0.81)	0.82 (0.80–0.85)	0.74 (0.71–0.78)	0.77 (0.73–0.80)
Peri-MI segments	**–**	**0.87** **(0.85–0.89)**	0.87 (0.86–0.87)	0.86 (0.84–0.91)	0.87 (0.80–0.87)	0.88 (0.87–0.90)
Contralateral segments	**–**	**0.88** **(0.85–0.90)**	0.88 (0.86–0.90)	0.87 (0.85–0.88)	0.88 (0.83–0.90)	0.90 (0.88–0.92)
Remaining segments	**–**	**0.87** **(0.83–0.90)**	0.85 (0.82–0.87)	0.89 (0.86–0.91)	0.87 (0.85–0.90)	0.87 (0.82–0.89)
Overall	**0.90** **(0.88–0.92)** p = 0.0001*	**0.85** **(0.80–0.89)** **p = 0.0001**†	0.85 (0.79–0.87)	0.86 (0.83–0.90)	0.85 (0.75–0.88)	0.85 (0.78–0.89)

Comparisons of MDI between segments were performed by a Kruskal–Wallis non-parametric test. All values are expressed as median with interquartile range.

*MI* myocardial infarction.

*p = 0.0001 for comparison between MDI in MI segments and peri-MI, contralateral, and remaining segments in comparison with MDI in control. Comparisons of MDI between segments were performed by a Kruskal–Wallis non-parametric test.

^†^*p* = 0.0001for comparison between MDI in MI, peri-MI, contralateral and remaining segments of post-MI hearts.

#### Vascular tree

**Figure 3:**
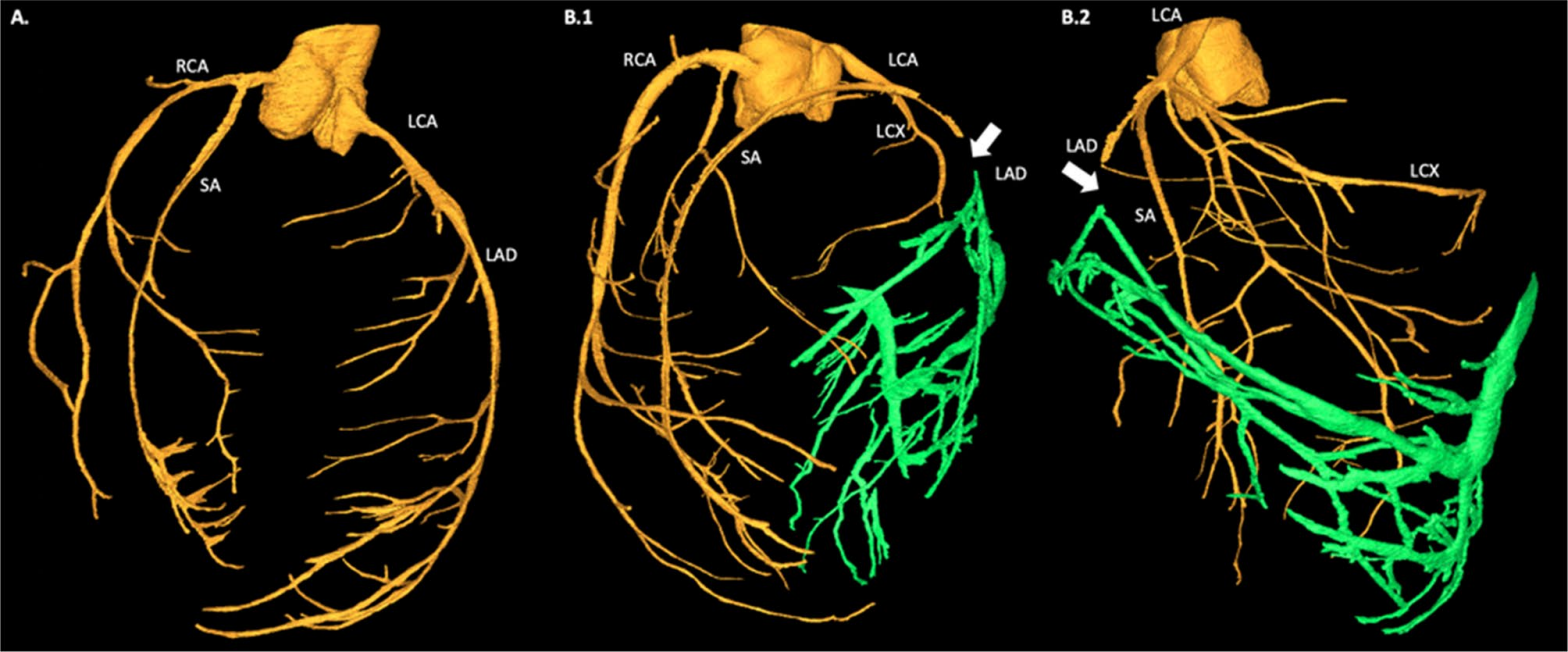
3D Rendering of the segmented vascular tree. For this rendering one control heart from a healthy rat, and one infarcted heart from a rat with induced myocardial infarction was used. (**A**) Control rat heart. (**B.1**, **B.2**) Post-myocardial infarction heart. Coronary arteries branching is represented in yellow. Green color indicates increased vascular spaces in the infarct area. White arrow indicates site of left coronary artery ligation. RCA: right coronary artery, LCA: left coronary artery, SA: septal artery, LCX: left circumflex artery, LAD: left anterior descending artery.

The segmentation and subsequent visualization of the vascular tree allowed to identify the precise location of left coronary artery occlusion. It also showed a lack of collateral circulation in the MI area, confirming the development of the transmural MI. On the other hand, it also showed increased vascular spaces in the infarct area that may represent various stages of angiogenesis and a basis for future (retrograde) collateral circulation formation (Fig. 3). Similar to some previous reports, the vascular spaces in the infarcted area seem larger, and with less defined vascular walls possibly indicating a venous origin or extensive branching processes of capillaries (Supplementary videos S1, S2, and S3)^17–19^.

### Mesoscale

#### Local tissue characterization

**Figure 4:**
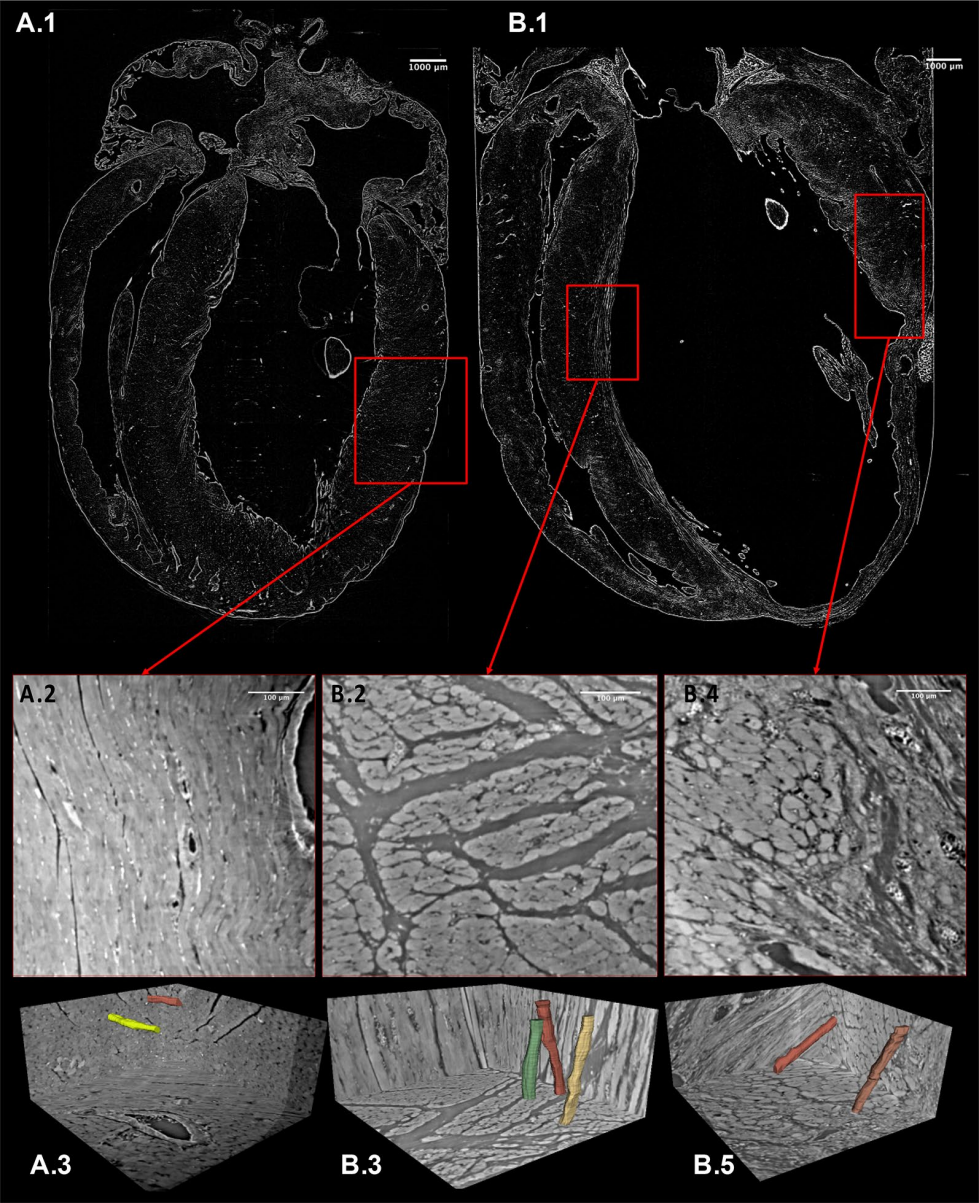
HR images and their location in the hearts. Upper panel: LR images of the control rat heart (**A.1**) and a rat heart following myocardial infarction (**B.1**), indicating the exact areas (red rectangles) from which HR images were taken. Middle panel: HR images from the selected areas, showing cardiomyocytes in both longitudinal and transversal directions. Lower panel: Three-dimensional sub-volumes of the HR datasets with segmented individual cardiomyocytes (manually delineated in 3D Slicer). HR—high resolution, LR—low resolution.

**Figure 5:**
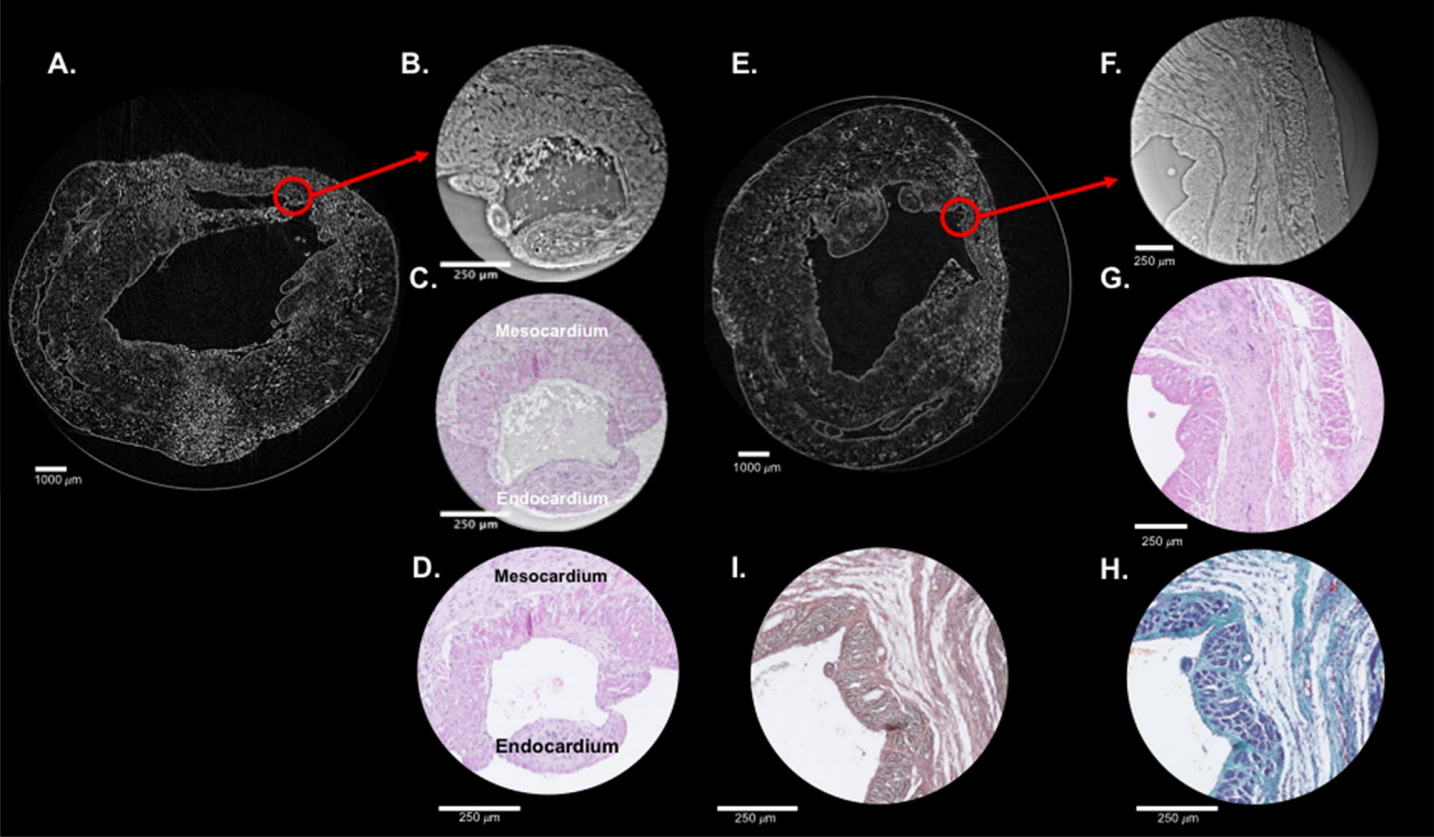
Comparing PB-XPCI *virtual histology* and conventional light microscopy. For this image one infarcted heart from a rat with induced myocardial infarction was used. Left panel-direct comparison of the identical myocardial area imaged by PB X-PCI *virtual histology* and light microscopy. (**A**) Low resolution PB X-PCI of the infarcted heart. Red circle indicates part of the infarcted area that was imaged by high resolution PB X-PCI. (**B**) High resolution PB X-PCI image of the selected area. (**C**) Overlap of the approximately same slice by PB X-PCI and light microscopy. (**D**) Hematoxylin and eosin-stained light microscopy. On every image from (**B**) to (**D**), a varying orientation of cardiomyocytes aggregates may be observed-at endocardial side longitudinally oriented; while towards the mesocardium are circumferentially oriented. Right panel (**E**) PB X-PCI image at low resolution representing the short axis view of the left ventricle and indicating the region of interest used for acquisition of the high-resolution PB X-PCI showed on (**F**). (**G**) Hematoxylin and eosin staining of the identical region of the same heart showing high resemblance to PB X-PCI virtual histology image. (**H**) Mallory's trichrome staining showing increased amounts of mature collagen (shown in blue color) in the area of myocardial infarction, i.e. scar tissue. (**I**) Gömöri's silver impregnation staining showing no reticular fibers in the region of interest (no areas stained in black on the sample).

Several specific myocardial segments were used for high resolution (HR) imaging, serving as a form of virtual histology, and compared to classical assessment. At the effective pixel size of 0.65 µm, and in the whole heart, with no further specimen preparation or staining, the cardiomyocytes, forming the local 3D myocardial micro-organization, could clearly be visualized (Fig. 4). The intercellular matrix, containing fibrous tissue and vasculature, was also easily identified. Figure 5 shows the comparison of the same myocardial area imaged by *virtual histology* PB X-PCI, and conventional histology. Besides close resemblance of the images produced by these two different techniques, the information content is similar. We may clearly observe the preserved longitudinally arranged cardiomyocytes on the endocardial side, and circumferentially arranged towards the mesocardium. Virtual histology obtained by PB X-PCI, due to its three-dimensionality, allows for easy tracking of entire cardiomyocytes bundles, providing information on its interrelation with the overall myocardial structure.

**Figure 6:**
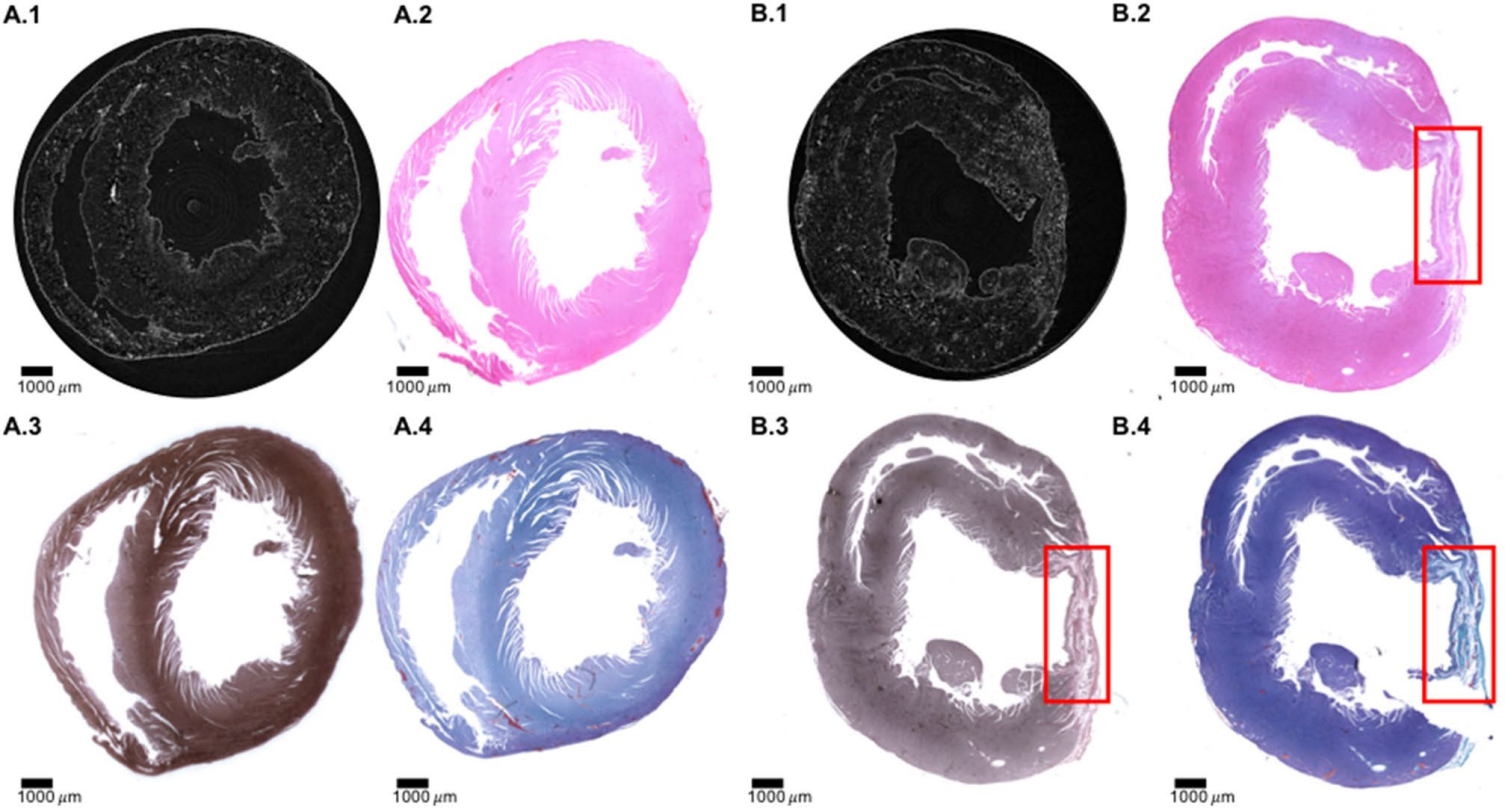
Comparison of PB X-PCI virtual histology and classical histology. One control heart from a healthy rat, and one infarcted heart from a rat with induced myocardial infarction was used. (**A.1–A.4**) Control rat heart in short axis view. (**A.1**) PB X-PCI virtual histology image. (**A.2**) Hematoxylin–eosin, (**A.3**) Gömöri's silver staining, (**A.4**) Mallory's trichrome of the same heart. (**B.1–B.4**) Myocardial infarction rat heart. (**B.1**) PB X-PCI virtual histology image. (**B.2**) Hematoxylin–eosin, (**B.3**) Gömöri's silver staining, (**B.4**) Mallory's trichrome of the same heart. Red boxes on (**B**) images indicate area of scar following myocardial infarction.

To illustrate the potential for tissue characterization, Fig. 6 compares the whole short-axis slice LR PB X-PCI versus different histology staining of the control and an infarcted heart.

Figure 5 (right panel) shows this in more detail comparing the LR and HR PB X-PCI image with histology at larger magnification.

In the early phases of an MI, reticular fibers (non-mature forms, rich in collagen type III) are usually present, representing the ongoing healing process. In our samples, reticular fibers could not be visualized, confirming that the scarring process of the infarcted areas has been finalized, and that true transmural scars are formed (Figs. 5, 6).

### Microscale

#### Quantification of cellular remodeling

The HR level achieved by PB X-PCI allowed identification and quantification of individual cardiomyocyte size. While very few cardiomyocytes were present in the MI zones, in all other regions cardiomyocyte hypertrophy was present, with larger average cross-sectional area (CSA) in the post-MI hearts in contrast to control rat heart (764 (640–810) μm^2^ (anterior wall) and 459 (410–530) μm^2^ (septum) vs. 296 (285–322) μm^2^ and 311 (275–318) μm^2^), indicating a significant difference between peri-MI and contralateral areas of post-MI hearts, as well as compared to the control rat heart (Table 1). Cardiomyocyte CSA were consistent between post-MI hearts 1 and 2 (Table1).

## Discussion

In this manuscript, we present a comprehensive assessment of the macro-, meso-, and microstructural changes caused by MI in an ex vivo rat model of left coronary artery ligation. PB X-PCI provides visualization and quantification of morphological changes on whole organ, tissue organization and type, vascular as well as individual cellular level, with a single, non-destructive 3D imaging modality, in the absence of further specimen manipulation, contrast agents or tissue staining.

The entirety of information gained by PB X-PCI in an animal model of ischemic heart disease would conventionally require using several different imaging modalities in conjunction with (destructive) histopathological analysis. Whole-heart morphology and quantification of cardiac remodeling is usually obtained by means of echocardiography, (micro-) computed tomography, or magnetic resonance imaging in in vivo research animal models or in clinical medicine^2,20^. For the analysis of changes at the cellular level, light and electron microscopy are the routine methods. While confocal or transmission microscopy offers some 3D characterization at the cellular level, imaging depth is still a major limitation and tissue preparation remains cumbersome^21^. Automated (ion-beam or others) slicing methods help, but only up to a certain point^22,23^. The major drawbacks of histological procedures therefore include time-consuming sample preparation, usually demanding destruction of tissue, as well as different staining methods. Additionally, the need for thin slices makes 3D assessment of the analyzed structure very difficult since the off-line reconstructions of 3D structures are often performed from 2D images, with problems with slice loss or distortion^21^.

In recent years, X-ray computed tomography using synchrotron radiation has emerged and has been validated for imaging of different cardiac pathologies by assessing native intact animal heart models, as well as ex vivo human fetal hearts, and transmural samples of adult human hearts, both normal and disease-affected (such as fetuses with congenital heart disease)^6–16^. For example, Gonzalez-Tendero et al. illustrated that PB X-PCI can quantify cardiac structures and tissue with enough details to reveal microfiber and vascular structure throughout whole rodent and rabbit fetal hearts, focusing on remodeling associated with intrauterine growth restriction^6,15^. They showed that the great advantage of PB X-PCI, as compared to micro-computed tomography (micro-CT), is that it provides a better contrast between soft cardiac tissues than classical X-ray absorption imaging.

In this study, we have shown that PB X-PCI allows for multiscale quantification of the precise regional and transmural extension of an MI, from organ to cellular level. At the macroscale, it offers the quantification of global cardiac remodeling, such as wall thickness, myocardial mass and cavity volumes, as well as the visualization of the coronary arterial and venous tree, enabling the precise identification of the location of the left coronary artery occlusion. While global remodeling in the setting of an MI is well understood, the whole heart macro-vascular assessment offered by PB X-PCI allows new avenues to explore. For example, the adaptive response and regeneration of the vasculature can be clearly seen from the increased vascular branching and the presence of enlarged thin wall vessels in the area of the MI. These large vessels were described before using histology and their overall effect studied by MRI, but PB X-PCI allows the direct visualization and quantification in the whole 3D infarct area where both the arterial as well as the venous branches can be studied without the need for contrast agents^24^. At HR, even some properties of the vascular walls can be appreciated.

At the mesoscale, specific magnetic resonance techniques, such as diffusion tensor imaging (DT-MRI) and tractography, have been used to characterize in vivo and ex vivo the transmural course of the orientation of cardiomyocyte aggregates (“*fibers*”) in 3D—an important component of cardiac architecture that might be affected by different cardiac conditions, such as MI. However, compared to the size of cardiomyocyte, DT-MRI has a limited resolution (with the smallest achieved pixel sizes many times larger than individual cardiomyocytes) and specifically assesses diffusivity in water-containing structures, which might not make it the ideal modality to investigate scarred or highly fibrotic tissue or when assessing intracellular matrix remodeling. Additionally, the visual representations of *fiber-tracks* obtained by tractography do not directly show all local properties and are affected by imaging artefacts^25,26^.

As opposed to DT-MRI, PB X-PCI, with a much better resolution and assessment of the non-watery structure, enables direct visualization, as well as local quantification, of cardiomyocyte aggregates and their orientation, thus providing insight to the local organization of the myocardium in detail.

From the same whole heart imaging setup, combined with our multiscale approach, PB X-PCI also enabled to study adaptations at the cellular level and assess cellular remodeling by quantifying hypertrophy of individual cardiomyocytes in different myocardial regions. Similarly to what was shown before from histopathological studies, we measure significant differential hypertrophy induced by the presence of an MI, both in the preserved myocardium adjoining the infarct, as well as in contralateral (distant) myocardial regions, however more pronounced in the former area^1^. Combining the different scales (as shown in Fig. 5) provides a novel, more integrated approach towards assessing infarct remodeling, its mechanisms and the potential therapeutic benefits. There is still some controversy about the formation of an infarct and the resulting tissue structure. Studies using (DT-) MRI, suggested a clear decrease of local organization and absence of “fiber”-directions in the infarct^27^. Others, using a different analysis approach, suggested that the global orientation of myocardial sheets is preserved^28^. These discrepancies likely originate from the principle of MRI, predominantly assessing the presence and dynamics of water molecules in cardiomyocytes and the intercellular space^29^.

Using classical histopathology approaches, it was shown that despite a large increase in collagen density, the organization is maintained in the core of infarction in both acute and chronic MI. While collagen is clearly synthesized in the infarction, its orientation (just like the initial ‘fiber’ directions during cardiac development) is influenced by mechanical loading direction^30–33^.

As can be seen from Fig. 2, our images as well as the quantitative regional ‘fiber’ analysis focused on small tissue volumes, show tissue organization in the infarct. While there is a small, but significant, difference in the MDI between regions affected by MI, peri-MI areas, and contralateral areas, as well as between MI-affected and control rat hearts, the overall organization is clearly preserved. Although only very few cardiomyocytes remain in the area of transmural MI, the intercellular meshwork is still similar in structure and show a clear preserved organization, with gradually changing direction from epi- to endocardium. This suggests that the lack of perfusion results in the disappearance of cardiomyocytes and a collapse of the matrix. Interestingly, both at the endocardial as well as epicardial border, a fully intact myocardial layer with a thickness of several cardiomyocytes remains present, likely thanks to the perfusion by diffusion from the blood in the cavity and the pericardial fluid.

In summary, in this paper we have shown that PB X-PCI offers a powerful research tool to study tissue remodeling following an MI using a whole heart, 3D, non-destructive, non-staining or contrast-based technique, feasible for the quantification of myocardial remodeling at whole-organ, tissue, vascular as well as cellular level in ex vivo model of ischemic heart disease.

## Limitations

Synchrotrons are large scale facilities with limited access due to high competition for the available time slots. Although this currently limits the amount of studies that can be performed, there is a clear technical development pathway towards using PB X-PCI based on laboratory X-ray sources or alternative laser-electron beam setups. Micro-CTs with X-PCI capabilities can be expected in the near future^34–40^.

Also, while studies on imaging beating hearts (Langendorff and in-vivo) with X-PCI are underway, the current research was performed on static, ex-vivo hearts thus lacking direct correlative function assessment, and lacking direct comparison with in vivo methods.

Limited number of animal models of disease and one control animal hinder the validation power of this study, however the aim was primarily to show the comprehensive potential of the method in the setting of ischemic heart disease pathological processes.

Finally, the current field-of-view of most synchrotron imaging beamlines only allows to study small (rodent and human fetal) specimens given that scan time importantly increases when stitched acquisitions need to be performed.

## Methods

### Animal model

Post-MI remodeling was recapitulated in a well-established rodent model. A transmural MI was induced by left coronary artery ligation through a left thoracotomy in 8 to 11 week-old Wistar male rats (Charles River Laboratories, France, EU)^41^. Rats were sacrificed after 2 weeks, the hearts were excised, and immersed in formalin. Rats were euthanized with an anaesthetic overload (5% inhaled isoflurane) and subsequent exsanguination. For this experiment 4 rats with induced MI, and 1 control healthy rat were used. Online Supplementary methods explain in more detail animal handling.

Animal care and experimentation conformed to the European Union (Directive 2010/63/UE) and Spanish guidelines (RD 53/2013) for experiments involving animals. This study was approved by the local animal research ethics committee “Comité de Ética de Experimentación Animal (CEEA)” (CEEA 68/5435). The study planning, design, implementation and reporting was performed in compliance with ARRIVE guidelines.

### Data acquisition

Synchrotron radiation-based X-PCI image acquisition was carried out at the TOMCAT beamline (X02DA) of the Swiss Light Source (Paul Scherrer Institute, Switzerland). The samples were placed in dedicated tubes with degassed deionized water as medium to reduce the chance of bubble formation. The samples were then placed on the stage for acquisition with a multi-scale PB X-PCI setup^7^. Briefly, the sample is fully illuminated by the X-ray beam and the microscope is placed at a certain distance from the sample in order to allow for interference patterns to form, which will induce PB contrast enhancement, especially on the interface between different tissues. In our case, a monochromatic beam with an energy of 20 keV and two configurations were used. First, a LR configuration is chosen to image the whole heart at 5.8 µm effective pixel size, with a sample-detector distance of 333 cm, using a LuAG:Ce 300 µm scintillator (Crytur, Czech Republic) and a PCO.Edge 4.2 CMOS detector (PCO AG, Kelheim, Germany). The resulting dataset is used for navigation of regions of interest to be imaged with a HR setup (at 0.65 µm effective pixel size, sample-detector distance of 20 cm, with a LuAG:Ce 20 µm scintillator (Crytur, Czech Republic) and a PCO.Edge 5.5 CMOS detector (PCO AG, Kelheim, Germany)), and for offline assessment of whole organ remodeling, cardiomyocytes orientation analysis, and vasculature visualization^7^.

When the sample is too large to fit fully in the field of view, several overlapping scans have to be acquired and stitched a posteriori. The acquired projections are reconstructed using the Gridrec algorithm both in absorption and applying the single distance phase retrieval method developed by Paganin^41–43^.

### Image analysis

Fiji, an open-source platform for biological-image analysis, was used to analyze and visualize gross anatomical features, as well as induced histopathological remodeling by myocardial infarction (ImageJ v.1.51 s, Wayne Rasband, National Institute of Health, USA)^44,45^.

Pre-processing of the LR datasets, such as contrast and brightness enhancement, was performed to ease the process of feature detection.

Then, a semi-automatic, whole heart segmentation was performed on the LR datasets using Ilastik to obtain first a rough segmentation, followed by smoothing by an in-house algorithm implemented in MATLAB (R2018b)^46^.

### Left ventricular myocardial and cavity volumes calculation

In order to calculate the LV myocardial and cavity volumes, the epicardial and endocardial borders of the LV were manually delineated in some short-axis image slices of the LR datasets from the apex to the base. Then a 3D interpolation was performed to obtain the whole LV mask using a custom algorithm implemented in MATLAB from which both LV myocardial and cavity volumes were quantified.

For the detailed regional analysis of structural myocardial changes, a 45-segment myocardial model was used^47^. Every segment was assessed for myocardial damage and categorized in three categories, defined as: 1. Transmural MI: the whole segment is scarred, 2. peri-MI: segments immediately adjacent to the infarcted area, with no signs of scarring, 3. contralateral segments: segments directly opposite to the infarcted ones, with no signs of loss of myocardium.

For each of the segments, LV myocardial thickness was also automatically calculated by computing the Euclidean distance transform of the binary LV mask using a custom algorithm implemented in MATLAB.

### Cardiomyocyte segmentation and cross-sectional area calculation

The high resolution achieved by PB X-PCI allowed identifying and analyzing individual cardiomyocytes in the HR datasets, that were segmented and their CSA was quantified using open-source software (Fiji-Image J and 3D Slicer)^48,49^.

In our cardiomyocyte analysis, we included two post-MI and the control hearts. Cardiomyocytes were analyzed in sub-volumes sized 551.2 µm × 551.2 µm × 260 µm (length × width × height) of the HR datasets. In post-MI hearts, cardiomyocytes in the area of infarction (including preserved cells adjoining the fibrotic post-MI tissue), as well as in the non-affected myocardium were analyzed. The infarcted area was part of the anterior wall, while the non-affected area analyzed was located in the septum. The same corresponding areas were also quantified in the control rat heart. Two sub-volumes were selected per area, one closer to the endocardium, and the other closer to the epicardium. Individual cardiomyocytes were segmented by manual delineation. Then CSA calculation was performed in Fiji-ImageJ and expressed as the median value with interquartile ranges of measurements of 10 cells per sub-volume.

### Cardiomyocyte aggregates orientation

The orientation of the local cardiomyocyte aggregates (often referred to as ‘fibers’) was assessed on LR datasets using a custom structure tensor (ST) based algorithm implemented in MATLAB^8,16^. Briefly, for each image voxel, the gradient in x, y and z directions was computed using a central difference algorithm. Then, the ST in each image voxel was obtained as the cross product of gradient vectors (g_x_, g_y_ and g_z_), which is in matrix notation:

$$ST=\left(\begin{array}{ccc}\sum{{g}_{x}^{2}}& \sum{{g}_{x}{g}_{y}}& \sum{{g}_{x}{g}_{z}}\\ \sum{{g}_{y}{g}_{x}}& \sum{{g}_{y}^{2}}& \sum{{g}_{y}{g}_{z}}\\ \sum{{g}_{z}{g}_{x}}& \sum{{g}_{z}{g}_{y}}& \sum{{g}_{z}^{2}}\end{array}\right).$$

Eigen-decomposition was applied to the ST to obtain the three eigenvectors (${\overrightarrow{\boldsymbol{v}}}_{\boldsymbol{i}})$ and their corresponding eigenvalues (λ_i_). The eigenvector with the smallest eigenvalue (tertiary eigenvector, ${\overrightarrow{\boldsymbol{v}}}_{3}$) was considered to be the one pointing in the longitudinal direction of cardiomyocytes as it corresponds to the direction with lowest image intensity variation (see Supplementary Figure S4). The HA, also known as inclination angle, was calculated for each image voxel as the angle between the tertiary eigenvector ${\overrightarrow{\boldsymbol{v}}}_{3}$ and the local circumferential plane, defined by the local longitudinal and circumferential directions of the cylindrical coordinates system of the heart (see Supplementary Figure S4). Additionally, cardiomyocyte aggregates orientation was also measured in HR dataset from the LV of the healthy heart.

Cardiomyocyte aggregates orientation analysis and quantification was performed across the 45 LV myocardial segments. Transmural profiles (from endo- to epicardium or right-side endocardium) of the HAs in all of the LV segments were obtained to see how HA of cardiomyocyte aggregates changes along the ventricular wall. Linear regression fitting of the transmural profiles of the HA was done (y = β_0_ + β_1_*x, where "y" corresponds to the HA, and "x" is the normalized wall depth, ranging from 0.0 in the endocardium to 1.0 in the epicardium), and the two regression coefficients β_1_ and β_0_, and the R^2^ (linearity) coefficient of the linear fitting were obtained. β_1_ represents the total HA gradient over the myocardial wall while β_0_ is the intercept term.

Local myocardial disarray was quantified by the calculation of the MDI as proposed recently by Garcia-Canadilla P et al.^9^ This index quantifies, for each image voxel, the uniformity of the cardiomyocytes’ longitudinal direction (represented by the tertiary eigenvector) within a neighborhood of size of 200 µm^3^. The MDI ranges between 0 and 1, with larger values indicating highly organized cardiomyocytes (insignificant disarray) whereas lower values of MDI denote a greater loss of cardiomyocytes organization (high degree of disarray) (see Supplementary Figure S4).

### Vasculature visualization

PB X-PCI images show enough tissue contrast to identify vessels without the need for X-ray contrast agents. On the LR datasets the ostia of the coronary arteries were identified at the level of aortic valve, and a detailed segmentation of main coronary arteries and its main branches was performed in 3DSlicer^48,49^. The resulting 3D coronary artery tree was visualized using Seg3D and used for the identification of the exact location of the left coronary artery occlusion^50^. Furthermore, the vascular branching was identified and visualized in the area of MI.

### Histopathology

Following the PB X-PCI imaging of the intact hearts, a ‘gold-standard’ histopathological analysis was performed for comparison with the PB X-PCI images, obtained at both the cellular and organ level. The hearts were cut, embedded in paraffin and sectioned with a microtome into 4 µm slices that were mounted on glass slides and stained using hematoxylin and eosin dyes. Furthermore, Mallory's trichrome and Gömöri's dyes were used to better discern mature collagen (presumably rich in collagen type I) and reticular fibers. The tissue slides were imaged using light microscopy with 0.55 × and 20 × magnification (approximately 2.2 and 0.7 µm point-to-point resolution respectively). Furthermore, images from glass slides were digitized using the microscope camera (microscope Zeiss, model AX10/Lab.A1/; camera AxiocamERe5s; acquisition program ZEN2.5) and NanoZoomer 2.0 RS (Hamamatsu Photonics, Japan).

### Statistical analysis

Normality of data was tested with a Shapiro-Wilks test, adequate for a small number of samples. Normally distributed continuous variables were expressed as means with standard deviation, while non-normally distributed continuous variables were expressed as medians with interquartile ranges. Comparison between two groups was done using a two-tailed Student’s T-test, while comparisons between multiple groups were done using a non-parametric Kruskal Wallis test. For all statistical tests the alpha level was determined at 0.05. Statistical analysis was performed using STATA (Stata/IC 13.1 for Mac, Statacorp, Texas, USA).

## Supplementary Information

Supplementary Information 1.

Supplementary Video 1.

Supplementary Video 2.

Supplementary Video 3.

## References

[1] Pfeffer MA, Braunwald EB (1990). Ventricular remodelling after myocardial infarction. Circulation.

[2] Cohn JN, Ferrari R, Sharpe N (2000). Cardiac remodelling-concepts and clinical implications: a consensus paper from an international forum on cardiac remodelling. On behalf of an International Forum on Cardiac Remodelling. J. Am. Coll. Cardiol..

[3] Heusch G (2014). Cardiovascular remodelling in coronary artery disease and heart failure. Lancet.

[4] Xie M, Burchfield JS, Hill JA (2013). Pathological ventricular remodelling: mechanisms: part 1 of 2. Circulation.

[5] Watson SR, Dormer JD, Fei B (2018). Imaging technologies for cardiac fiber and heart failure: a review. Heart Fail. Rev..

[6] Gonzalez-Tendero A (2017). Whole heart detailed and quantitative anatomy, myofibre structure and vasculature from X-ray phase-contrast synchrotron radiation-based micro computed tomography. Eur. Heart J. Cardiovasc. Imaging.

[7] Dejea H (2019). Comprehensive analysis of animal models of cardiovascular disease using multiscale X-ray phase contrast tomography. Sci. Rep..

[8] Garcia-Canadilla P (2018). Complex congenital heart disease associated with disordered myocardial architecture in a midtrimester human fetus. Circ. Cardiovasc. Imaging.

[9] Garcia-Canadilla P (2019). Myoarchitectural disarray of hypertrophic cardiomyopathy begins pre-birth. J. Anat..

[10] Mirea, I. *et al.* Very high-resolution imaging of post-mortem human cardiac tissue using X-ray phase contrast tomography. In *Functional Imaging and Modeling of the Heart*. 172–179 (Springer, 2015).

[11] Varray F (2017). Extraction of the 3D local orientation of myocytes in human cardiac tissue using X-ray phase-contrast microtomography and multi-scale analysis. Med. Image Anal..

[12] Kaneko Y (2017). Intact imaging of human heart structure using X-ray phase-contrast tomography. Pediatr. Cardiol..

[13] Tsukube T (2015). Impact of synchrotron radiation-based X-ray phase-contrast tomography on understanding various cardiovascular surgical pathologies. Gen. Thorac. Cardiovasc. Surg..

[14] Shinohara G (2016). Three dimensional visualization of human cardiac conduction tissue in whole heart specimens by high-resolution phase-contrast CT imaging using synchrotron radiation. World J. Pediatr. Congenit. Heart Surg..

[15] Dejea, H. *et al*. Microstructural analysis of cardiac endomyocardial biopsies with synchrotron radiation-based X-ray phase contrast imaging. In *Functional Imaging and Modeling of the Heart*. 23–31 (Springer, 2017).

[16] Baličević, V. *et al.* Assessment of myofiber orientation in high resolution phase-contrast CT images. In *Functional Imaging and Modeling of the Heart*. 111–119 (Springer, 2015).

[17] Dube KN (2017). Recapitulation of developmental mechanisms to revascularize the ischemic heart. JCI Insight.

[18] Del Monte G, Harvey P (2012). An endothelial contribution to coronary vessels. Cell.

[19] Das S (2018). A unique collateral artery development program promotes neonatal heart regeneration. Cell.

[20] Konstam MA, Kramer DG, Patel AR, Maron MS, Udelson JE (2011). Left ventricular remodeling in heart failure. Current concepts in clinical significance and assessment. JACC Cardiovasc. Imaging.

[21] Seidel T, Edekmann JC, Sachse FB (2016). Analysing Remodelling of Cardiac Tissue: a comprehensive approach based on confocal microscopy and 3D reconstructions. Ann. Biomed. Eng..

[22] Sands GB (2005). Automated imaging of extended tissue volumes using confocal microscopy. Micro Res. Technol..

[23] Pinali C, Kitmitto A (2014). Serial block face scanning electron microscopy for the study of cardiac muscle ultrastructure at nanoscale resolutions. J. Mol. Cell Cardiolog..

[24] Wang J (2014). Pathological mechanism for delayed hyperenhancement of chronic scarred myocardium in contrast agent enhanced magnetic resonance imaging. PLoS ONE.

[25] Naumova AV, Yarnykh VL (2014). Assessment of heart microstructure. From mouse to man. Circulation.

[26] Froeling M, Strijkers GJ, Nederveen AJ, Chamuleau SA, Luijten PR (2014). Diffusion tensor MRI of the heart-in vivo imaging of myocardial fiber architecture. Curr. Cardiovasc. Imaging Rep..

[27] Wu MT (2006). Diffusion tensor magnetic resonance imaging mapping the fiber architecture remodelling in human myocardium after infarction: correlation with viability and wall motion. Circulation.

[28] Geerts-Ossevoort L, Bovendeerd P, Prinzen F, Arts T, Nicolay K (2001). Myofiber orientation in the normal and infarcted heart, assessed with MR-diffusion tensor imaging. Comput. Cardiol..

[29] Bernus O (2015). Comparison of diffusion tensor imaging by cardiovascular magnetic resonance and gadolinium enhanced 3D image intensity approaches to investigation of structural anisotropy in explanted rat hearts. J. Cardiovasc. Magn. Reson..

[30] Hervas A (2016). Inhomogeneity of collagen organization within fibrotic scar after myocardial infarction: results in a swine model and in human samples. J. Anat..

[31] Whittaker P, Boughner DR, Kloner RA (1989). Analysis of healing after myocardial infarction using polarized light microscopy. Am. J. Path..

[32] Goergen CJ, Chen HH, Sakadžić S, Srinivasan VJ, Sosnovik DE (2016). Microstructural characterization of myocardial infarction with optical coherence tractography and two-photon microscopy. Physiol. Rep..

[33] Zimmerman SD, Karlon WJ, Holmes JW, Omens JH, Covell JW (2000). Structural and mechanical factors influencing infarct scar collagen organization. Am. J. Physiol. Heart Circ. Physiol..

[34] Vila-Comamala J (2018). Development of laboratory grating-based X-ray phase contrast microtomography for improved pathology. Microsc. Microanal..

[35] Viermetz MP (2018). High resolution laboratory grating-based x-ray phase-contrast CT. Sci. Rep..

[36] Otendal M, Tuohima A, Vogt U, Hertz HM (2008). A 9keV electron-impact liquid-gallium-jet x-ray source. Rev. Sci. Instrum..

[37] Reichardt M, Topperwien M, Khan A, Alves F, Salditt T (2020). Fiber orientation in a whole mouse heart reconstructed by laboratory phase-contrast micro-CT. J. Med. Imaging.

[38] Nakajima K (2008). Compact X-ray sources: towards a table-top free-electron laser. Nat Phys..

[39] Schlenvoigt HP (2007). A compact synchrotron radiation source driven by a laser-plasma wakefield accelerator. Nat. Phys..

[40] Eggl E (2015). X-ray phase-contrast tomography with a compact laser-driven synchrotron source. Proc. Natl. Acad. Sci..

[41] Cardin S (2012). Role for MicroRNA-21 in atrial profibrillatory fibrotic remodeling associated with experimental post-infarction heart failure. Circ. Arrhythm. Electrophysiol..

[42] Marone F, Stampanoni M (2012). Regridding reconstruction algorithm for real-time tomographic imaging. J. Synchrotr. Radiat..

[43] Paganin D, Mayo SC, Gureyev TE, Miller PR, Wilkins SW (2002). Simultaneous phase and amplitude extraction from a single defocused image of a homogeneous object. J. Microsc..

[44] Schindelin J (2012). Fiji: an open-source platform for biological-image analysis. Nat. Methods.

[45] Schindelin J, Rueden CT, Hiner MC, Eliceiri KW (2015). The ImageJ ecosystem: an open platform for biomedical image analysis. Mol. Reprod. Dev..

[46] Berg S (2019). Ilastik: interactive machine learning for (bio)image analysis. Nat. Methods.

[47] Captur G (2016). The embryological basis of subclinical hypertrophic cardiomyopathy. Sci. Rep..

[48] Kikinis R, Pieper SD, Vosburgh K, Jolesz FA (2014). 3D Slicer: a platform for subject-specific image analysis, visualization, and clinical support. Intraoperative Imaging and Image-Guided Therapy.

[49] Fedorov A (2012). 3D Slicer as an image computing platform for the quantitative imaging network. Magn. Reson. Imaging.

[50] Centre for Integrative Biomedical Computing at the University of Utah Scientific Computing and Imaging Institute. Seg3D: Volumetric Image Segmentation and Visualization; 2016. v2.4.4. http://www.seg3d.org.

