# Supplementary material for: Comprehensive assessment of myocardial remodeling in ischemic heart disease by synchrotron propagation based X-ray phase contrast imaging
Source: Sci Rep. 2021 Jul 7;11:14020. doi: 10.1038/s41598-021-93054-6 (PMC8263575; doi:10.1038/s41598-021-93054-6)
Supplement: Supplementary file 1 — Supplementary Information 1. [file 41598_2021_93054_MOESM1_ESM.docx]

Comprehensive Assessment of Myocardial Remodeling in Ischemic Heart Disease by Synchrotron Propagation Based X-Ray Phase Contrast Imaging

Ivo Planinc, MD^1*^, Patricia Garcia-Canadilla, PhD^2,3*^, Hector Dejea^4,5*^, Assoc. prof. Ivana Ilic, MD, PhD^6^, Eduard Guasch, MD, PhD^2,7,8^, Monica Zamora, MD, PhD^2,3^, Assoc. prof. Fàtima Crispi, MD, PhD^2,3,9^, prof. Marco Stampanoni, PhD^4,5^, prof. Davor Milicic, MD, PhD^1^, prof. Bart Bijnens, MD, PhD^2,10^, Anne Bonnin, PhD^4^, prof. Maja Cikes, MD, PhD^1^

**Expanded methods:**

*Animal models*

A transmural myocardial infarction was induced by left coronary artery ligation through a left thoracotomy in 8 to 11 week-old Wistar male rats (Charles River Laboratories, France, EU) with weight range of 150-200g. Rats continuously ECG-monitored were induced to anaesthesia with inhaled isoflurane 5%, and maintained with inhaled isoflurane 2% with concomitant analgesia by subcutaneous buprenorphine (0.05 mg/body kg), and were intubated and mechanically ventilated (SAR-1000, CWE Inc). Mechanical ventilation parameters were set according to the manufacturer instructions, including a 2 cmH_2_O external PEEP. The surgical procedure of thoracotomy consisted of incision through the left 4^th^ intercostal space, careful dissection through the tissue and the left coronary artery was exposed at its proximal aspect, in the interventricular septum close to the left atrial appendage. The artery was ligated with a 6-0 silk; anterior wall myocardial paleness and akinesia were observed immediately during the procedure in all animals, later followed by overt ST-segment elevation in a DI-like lead. Subsequently, the chest was closed with a 2-0 silk, and the skin with a 3-0 resorbable suture. Recruiting maneuvers were performed before extubation.

Control healthy rat was also a male Wistar rat from the same laboratory, and in the same weight range.


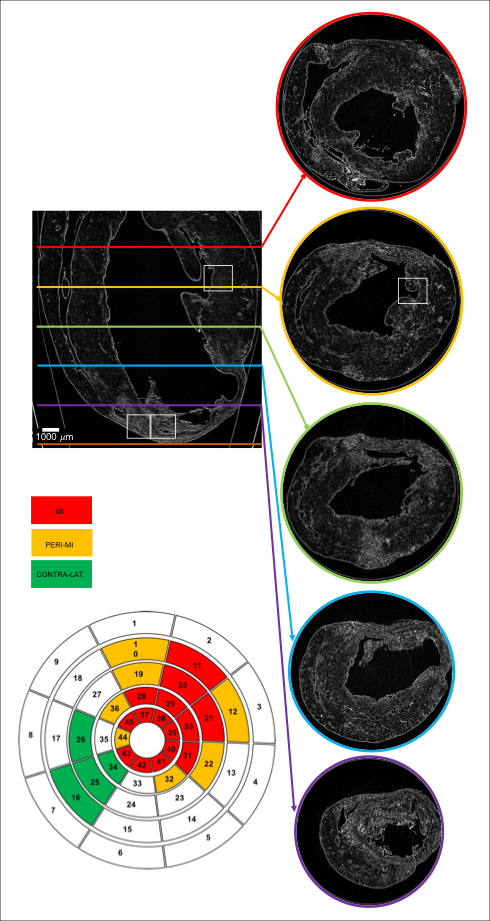


Supplementary Figure S1: An example of the 45-segment LV model of a heart with MI. One infarcted heart from a rat with induced myocardial infarction was used. A long axis view of the original PB X-PCI LR dataset as well as the five short axis images, used for assessment of specific LV segments, are shown. The white rectangles on the images indicate areas that were also imaged in high resolution and in which cellular features were analyzed.


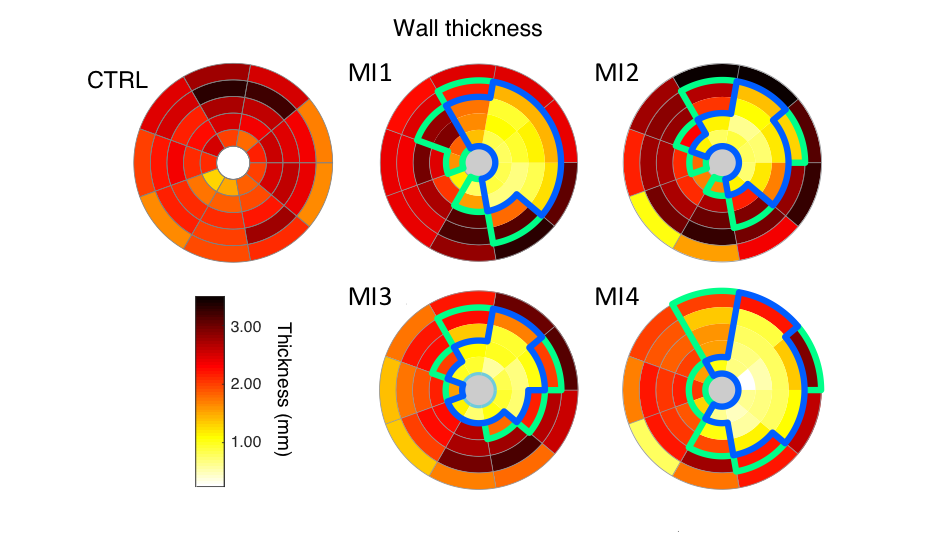


Supplementary Figure S2. Schematic representation of the 45-segment model visualizing regional wall thickness of LV rat hearts affected by a myocardial infarction as compared to the control. For this figure, 5 rat hearts were used (control heart from the healthy rat, and 4 infarcted hearts from 4 rats with induced myocardial infarction). The segments corresponding to MI (blue), and peri-MI areas (green) are highlighted.

CTRL: control rat heart; MI 1-4: post-MI rat hearts


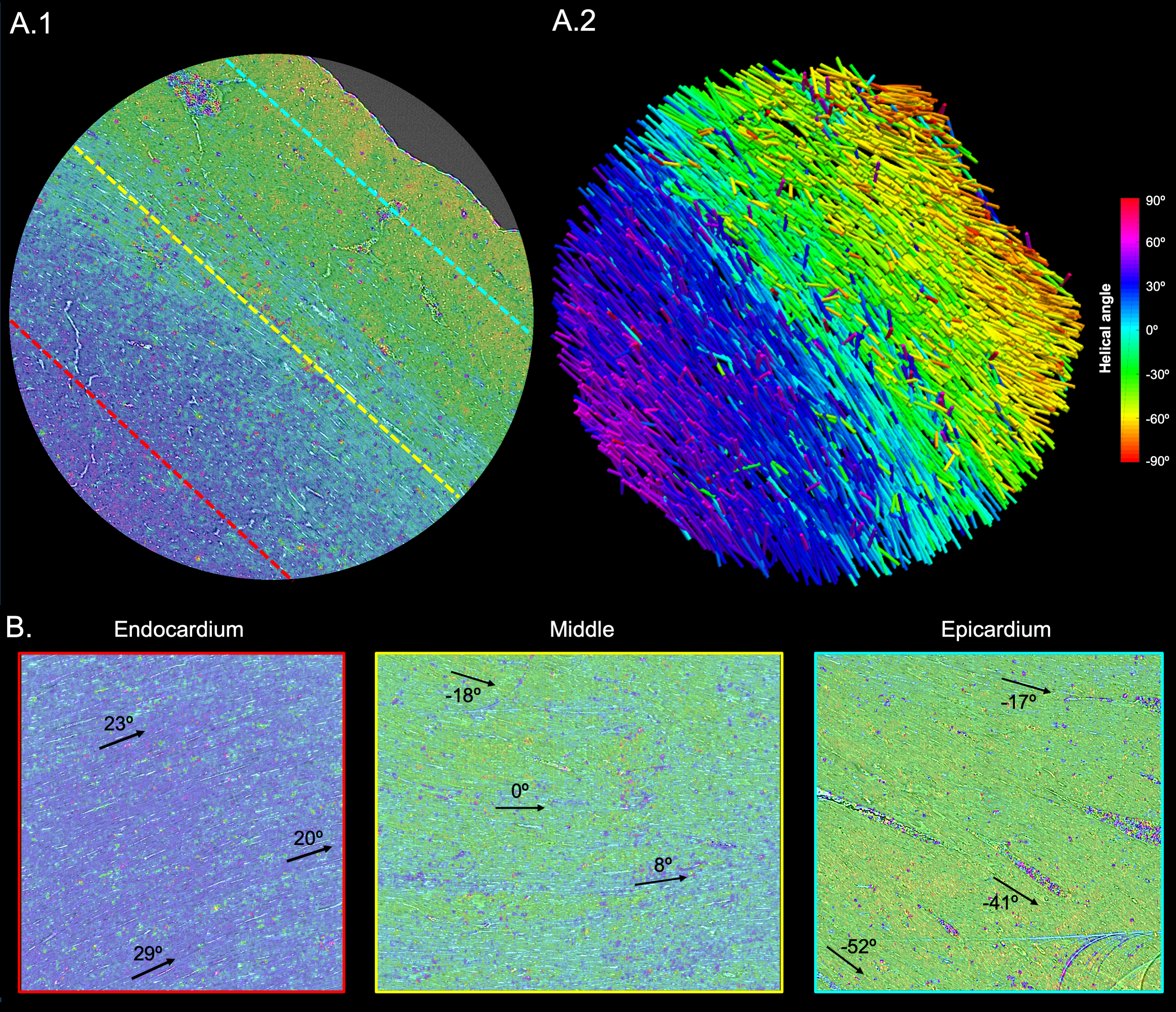


Supplementary Figure S3. A.1 Single X-PCI image slice of the HR dataset from the left ventricle (LV) of the healthy rat with its corresponding estimated local helical angle (HA) plotted in top. Different colors represent different HAs. A.2 3D representation of the principal vector (tertiary eigenvector ${\vec{\boldsymbol{v}}}_{\boldsymbol{3}}$) pointing in the long axis of cardiomyocytes for the same image slice represented in A.1. B. Estimated local helical angle (HA) plotted on top of their corresponding X-PCI image slices at the different transmural depth of the LV. The location of the three images within the LV wall are represented with dash-lines in A.1


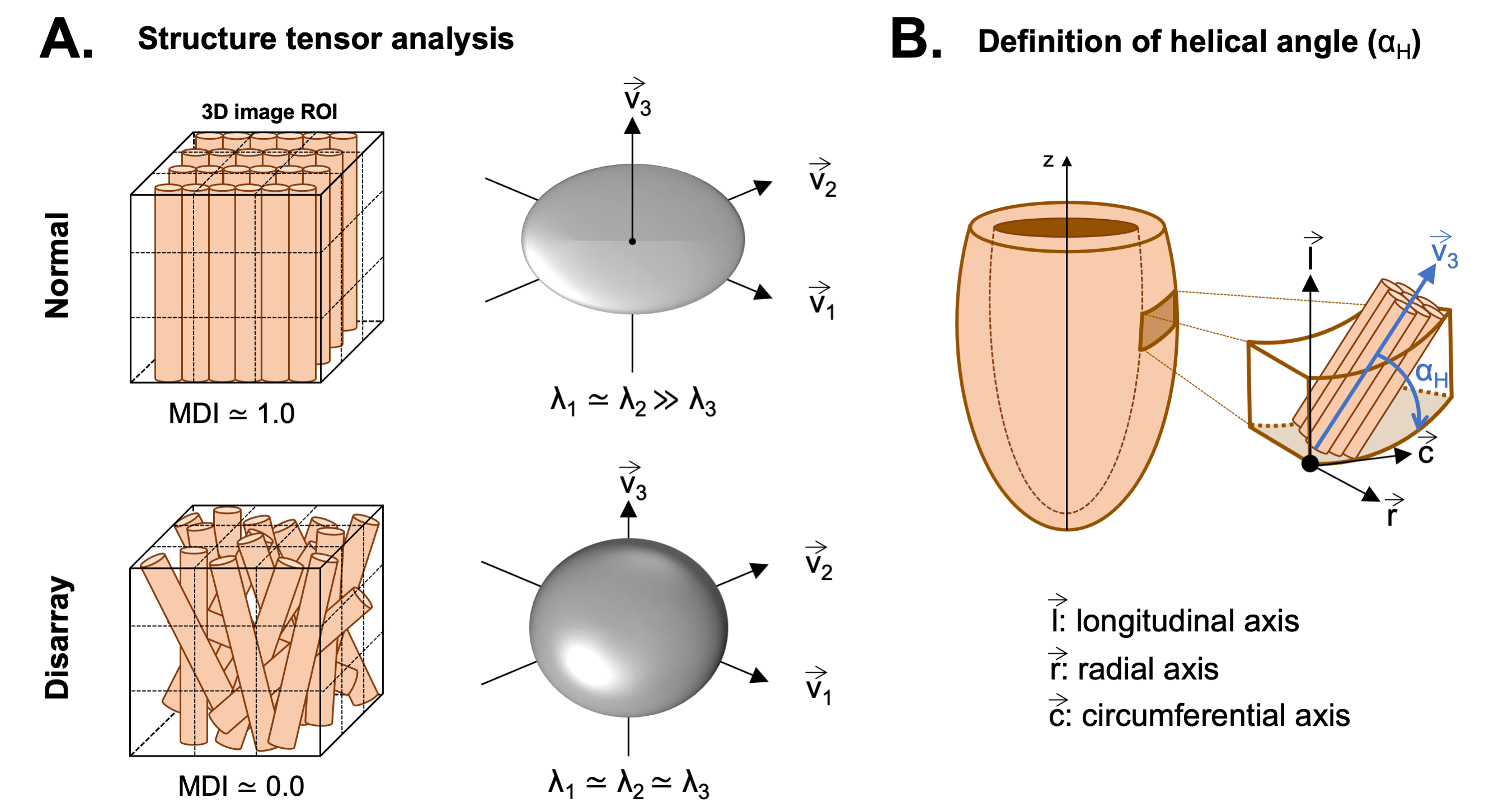


Supplementary Figure S4. Schematic representation of calculation of the helical angle (HA) of cardiomyocytes aggregates using the structure-tensor approach. A. Definition of the region of interest (ROI) defined to calculate the structure tensor in a given voxel, together with the eigenvectors system (${\vec{\boldsymbol{v}}}_{\boldsymbol{i}}$ = 1 … 3) and their ellipsoids obtained with structure tensor analysis in normal and in disarray conditions. B. Drawing deﬁning the local cylindrical coordinates of the left ventricle (LV). HA, denoted aH, is deﬁned as the angle between the local short-axis or circumferential plane and the tertiary eigenvector ${\vec{\boldsymbol{v}}}_{\boldsymbol{3}}$.

|  | **Control** | | | **Post-MI** | | |
| --- | --- | --- | --- | --- | --- | --- |
|  | **ß_0_** | **ß_1_** | **R^2^** | **ß_0_** | **ß_1_** | **R^2^** |
| **MI segments** | **-** | **-** | **-** | **91.1±26.8** | **-94.5**  **(-126.6 - -67.8)** | **0.82**  **(0.72-0.90)** |
| **Peri-MI segments** | **-** | **-** | **-** | **99.6±20.7** | **-60.1**  **(-81.0 - -52.1)** | **0.94**  **(0.89-0.97)** |
| **Contralateral segments** | **-** | **-** | **-** | **84.4±12.5** | **-50.2**  **(-60.6 - -41.5)** | **0.96**  **(0.93-0.97)** |
| **Remaining segments** | **-** | **-** | **-** | **88.1±28.3** | **-42.3**  **(-64.5 - -33.1)** | **0.95**  **(0.88-0.96)** |
| **Overall** | **77.7±18.1**  **p=0.0008*** | **-44.1**  **(-53.3 - -39.1)**  **p=0.0001*** | **0.95**  **(0.91-0.96)**  **p=0.0001*** | **90.8±25.1** | **-60.1**  **(-86.6 - -43.1)** | **0.93**  **(0.84-0.96)** |

Supplementary Table S1: Slope (β_1_), intercept (β_0_), and linearity coefficient (R^2^) of the linear fitting (y = β_0_ + β_1_*x) of the transmural profile of the HA in all the 45 segments.

*comparison between healthy rat heart and post-MI hearts. Comparison of β_1_, β_0_, and R^2^ between segments was performed by Kruskal-Wallis non-parametric test.

Normally distributed continuous variables are presented as means with standard deviations, and non-normally distributed as medians with interquartile range.

MI- myocardial infarction.

**Legends for video files**

Supplementary Video S1: Coronary anatomy of a control normal rat heart. One control heart from a healthy rat was used.

Supplementary Video S2: Coronary anatomy of a post-myocardial infarction rat heart, including post-ligation vascular spaces. One infarcted heart from a rat with induced myocardial infarction was used.

Supplementary Video S3: Superposition of 3-dimensional representation of post-ligation vascular spaces of a post-myocardial infarction rat heart on PB X-PCI images of the left ventricle in short axis view. One infarcted heart from a rat with induced myocardial infarction was used.
